# Psychosocial support interventions for improved adherence and retention in ART care for young people living with HIV (10–24 years): a scoping review

**DOI:** 10.1186/s12889-020-09717-y

**Published:** 2020-12-01

**Authors:** Emeka F. Okonji, Ferdinand C. Mukumbang, Zaida Orth, Shelley A. Vickerman-Delport, Brian Van Wyk

**Affiliations:** School of Public Health, University of the Western Cape, P Bag X17 , Bellville, Cape Town, 7535 South Africa

**Keywords:** Adolescents, Psychosocial support, Interventions, HIV and AIDS, Adherence and retention

## Abstract

**Background:**

Mental health disorders such as high levels of anxiety, isolation, depression and suicide ideation reported among young people living with HIV (10–24 years;YPLHIV) contribute significantly to poor medication adherence and retention in care. While there is evidence supporting the role of psychosocial support interventions in promoting adherence and retention in antiretroviral treatment (ART) among adults living with HIV, there is little evidence on the role of psychosocial support on medication adherence among YPLHIV. This scoping review was designed to identify and classify the types and effects of psychosocial support interventions designed to improve adherence and retention in ART among YPLHIV globally.

**Method:**

We searched six electronic databases (i.e., Scopus, Pubmed and EBSCOHost (Academic Search Premier, CINAHL, Psycarticles and Medline). Six relevant articles published between 2011 and 2019 met our inclusion criteria. We extracted information relevant to the nature and outcomes of the reported interventions using thematic content analysis informed by the Population, Intervention, comparison, outcome, and time (PICOT) framework.

**Results:**

Four distinctive treatment modalities that focused on improving ART adherence and retention in care were identified: individual counselling, support groups, family-centered services, and treatment supporters.

**Conclusion:**

There is a dearth of psychosocial support interventions to improve adherence and retention in ART amongst adolescents and young adults living with HIV. Future research and programming should seek to address psychosocial support interventions or approaches specifically designed to address the needs of YPLHIV.

**Trial registration:**

PROSPERO: Registration CRD42018105057.

## Background

Since the introduction of antiretroviral therapy (ART), significant gains have been made in mitigating the impact of the HIV/AIDS pandemic [1]. The increasing effectiveness of and access to ART, along with increasing innovations in ART service delivery have redefined the HIV epidemic from a deadly infectious disease to a chronic, manageable disease [1–3]. However, poor adherence to treatment and suboptimal retention in care continue to present significant challenges to ending AIDS by 2030 [4].

In 2018, UNAIDS estimated that 1.6 million young people aged 10–24 years were living with HIV [5, 6]. Therefore, young people living with HIV (YPLHIV) constitute a growing and key sub-population of people living with HIV globally. The increasing availability and effectiveness of ART worldwide has resulted in more children and adolescents living longer with HIV [7, 8]. However, it is well-documented that adolescents struggle to initiate, remain engaged, and consistently adhere to ART [9, 10]. While most of the individual, social and health systems barriers associated with ART adherence and retention in care affecting the general population also apply to YPLWH, the latter face greater risks of mental and behavioural health problems, which constitute additional barriers [7, 11, 12]. Psychological risk factors such as anxiety and depressive disorders result from the chronicity of HIV infection, being orphaned, changes of guardianship, and the nature of parental and other adult support [11, 13, 14].

Due to the high levels of anxiety, isolation, depression and suicide ideation reported among YPLWH, studies have recommended psychosocial support for YPLWH in addition to standard ART services to help them adapt and cope with the chronicity and stigma associated with HIV [3, 11, 12, 15–17]. Psychosocial support interventions are interpersonal or informational activities, strategies or techniques that can target biological, behavioural, cognitive, emotional, interpersonal, social or environmental factors with the aim of improving an individual’s health functioning and mental well-being [18]. To promote ART adherence and retention in care among YPLWH, a comprehensive psychosocial intervention is needed. Such psychosocial support interventions should promote HIV disclosure and communication, support adherence to medication, address feelings of isolation and other emotional-related distress, and the needs associated with emerging sexuality [19, 20].

Interventions such as counselling, cognitive behavioural therapy, and peer support have been applied to improve the mental health and overall well-being of people living with HIV over 18 years with success [21], supporting the role of psychosocial support interventions in promoting adherence and retention in ART care among adults living with HIV [22]. Nevertheless, there is little evidence on the nature and role of psychosocial support for YPLHIV [23]. To this end, in this review, we sought to identify, classify and assess the types and effects of psychosocial support interventions focused on improving adherence and retention in care among YPLHIV on ART in the current existing literature.

## Method

Our scoping review was conducted in line with the guidelines proposed by Khan et al. [24] i.e. (1) Framing the question; (2) Identifying relevant publications; (3) Assessing study quality; (4) Summarising the evidence; and (5) Interpreting the findings.

Based on the literature, we developed Boolean phrases that were tested using PubMed. The first literature search was conducted between March and October 2018. Due to unforeseen delays, an updated search using the same Boolean phrases and databases was conducted between October 2019 to March 2020. We searched multiple electronic databases – Scopus, PubMed and EBSCOHost (Academic Search Premier, CINAHL, Psycarticles and Medline) using a standard Boolean combination: “((adolescen* OR teenage* OR young people OR youth) [AND] (psychosocial intervention) [AND] (adherence in antiretroviral therapy OR retention in care))”. In addition, we hand-searched grey literature on mental health among YPLWH and transitioning YPLWH from paediatric to adult care. All titles and abstracts (including conference abstracts) were independently screened by SAV and ZO using the PICOT (Participants, Interventions, Comparisons, Outcomes and Time) mnemonics criteria described in Table 1. Discrepancies were resolved via discussions with a third researcher (FCM). Full texts of potentially relevant articles were retrieved and independently examined by the authors. The reference lists of considered relevant articles were also hand searched to identify further potentially relevant studies. Summaries of the interventions described in each article were retrieved using a standardized form, and key information such as study purpose, nature of intervention described, outcome of intervention and conclusions of each study were extracted.

**Table 1:** PICOT based inclusion criteria

Patient population	Adolescents or young adults (10–24 years) living with HIV
Intervention of Interest	Psychosocial support
Comparison interventions	None
Outcomes	
*Primary outcomes*	(1) Adherence to antiretroviral treatment (viral load); (2) Retention in care
*Secondary outcomes*	(1) Quality of life and wellbeing; (2) Stigma and discrimination; (3) Disclosure
Time	2005–2020
Other considerations	
*Language*	English

The acronym PICOT informed the eligibility criteria for inclusion in the scoping review: the population (participants) of focus, types of interventions (and comparisons), and the outcomes of interest. The time relates to the period within which the studies were published [see Table 1 below].

Studies were considered eligible for inclusion in this scoping review if they met the following criteria: (i) Evaluated the effects of or associations between psychosocial support intervention and adherence ART or retention in care or related biomedical outcomes e.g., viral suppression (primary outcomes). (ii) Reported quantitative measures of the primary outcomes. (iii) Targeted or included samples of YPLHIV (10-24) in a mixed sample. (iv) Was published between January 1, 2005 and March 31, 2020. Only articles published in English were considered. There was no restriction by geographical location.

Studies were excluded if they met the following criteria: (i) Adopted a qualitative research design (ii) Were a study protocol, or any form of review or conference abstracts not developed into full manuscripts. (iii) The population deviated from the age range specified. (iv) The intervention did not target the psychosocial needs of the study population. (v) The intervention focused on HIV prevention.

The quality of the included articles was rated as either “poor”, “fair” or “good” by three independent researchers (EO, SAV and ZO), and EO made the final adjudication in cases of non-agreement. The rating of the articles was based on the criteria provided by the NIH-NHLBI Quality Assessment of Systematic Reviews and Meta-Analyses assessment tool [25].

### Data extraction

The data were extracted using an excel spreadsheet under the following headings: study setting, sample characteristics, intervention objectives, study design and methods, outcome measures and results [Additional file 1].

### Data analysis

We employed a thematic content analysis approach to distil information from the selected articles [26]. The extracted information was coded into two broad categories: Intervention components and outcomes measured as informed by the study aim. The intervention components were coded/classified along the following categories: (1) How the interventions were administered; (2) who delivered the intervention; (3) Point of intervention delivery; and (4) components of the intervention. The outcomes were coded according to the reported primary and secondary outcomes of the study.

## Results

**Fig. 1:**
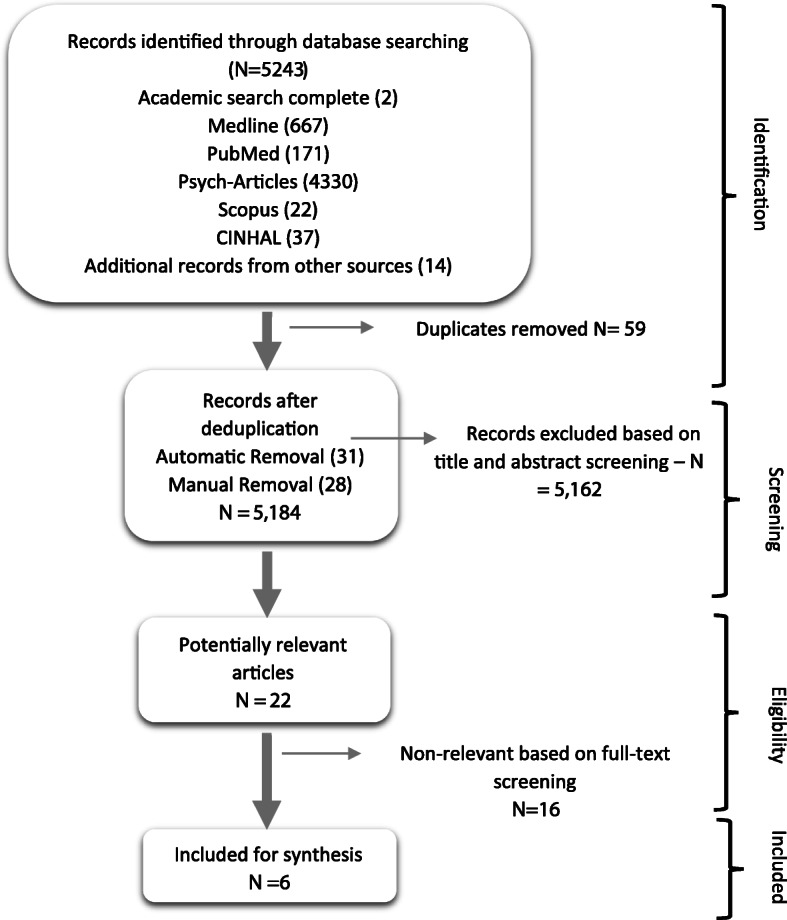
The PRISMA flow protocol to studies selection

Figure 1 shows the PRISMA diagram illustrating the selection process of the included studies. The literature search resulted in 5244 citations (Fig. 1), which were imported into a reference manager. Electronic (31) and manual (28) deduplication identified 59 duplicates. After screening for potentially relevant titles and abstracts 5162 articles were excluded. After screening full-texts, 17 papers were further excluded because they did not report on the effects of a psychosocial support intervention on adherence and retention in ART for YPLWH. Subsequently, six papers were included in the review of having good quantitative standards. Five studies were considered to be of good methodological quality [27–31] and one of a fair quality [32].

### Characteristics of included studies

The characteristics of the studies included in the review are summarised in Table 2.

**Table 2:** Characteristics of Included Studies (*N* = 6)

Characteristics	Count	References
Year of Publication		
2011–2015	3	[27, 28, 30]
2016–2020	3	[29, 31, 32]
Country		
United States of America	2	[28, 30]
Kenya	1	[29]
Uganda	1	[32]
South Africa	1	[27]
Zimbabwe	1	[31]
Study design (sample size)		
Pre and post intervention studies (61; 952)	2	[29, 30]
Randomized controlled trial (4504; 66; 94)	3	[27, 31, 32]
Retrospective cohort (174)	1	[28]

The six papers were disseminated between 2011 and 2019. Two of the studies were conducted in United States of America [28, 30] and four in Southern and Eastern Africa (Uganda, Kenya, South Africa and Zimbabwe) [27, 29, 31, 32]. Two of the papers were pre- and post-intervention studies (*n* = 1113) [29, 30], three randomized control trials (RCT) [27, 31, 32] (*n* = 4664), and one retrospective cohort study (*n* = 174) [28].

### Intervention duration

The duration of study was between 3 months and 10 years [Table 3].

**Table 3:** Intervention/study location and duration of intervention

Article	Country (location)	Duration of intervention
Wohl et al. [30]	USA (Los Angeles)	2 years
Davila et al. [28]	USA (Texas)	Decentralised era: 2 years Centralised area: 3 years Enhanced youth services: 10 years
Ruria et al. [29]	Kenya (Homa Bay)	6 months
Graves et al. [32]	Uganda	6 months
Bhana et al. [27]	South Africa (KwaZulu-Natal)	3 months
Willis et al. [31]	Zimbabwe (Gokwe south district)	12 months

Six studies evaluated psychosocial support interventions, namely: psychosocial education, group adherence counselling, individual counselling and peer-support groups and peer counselling. Two studies evaluated the impact of a family-centred appointment scheduling and health education on patient retention and adherence to monthly appointment scheduling [27, 32]. Three studies evaluated a youth centred management model that combined psychosocial case management, treatment education/adherence support and HIV risk reduction counselling to provide a client-centred intervention through which care was coordinated [28, 30, 31]. Three studies [29, 30, 32] evaluated interventions that included fast-track service deliveries to streamline medication pick-up. Table 4 illustrates the nature and characteristics of the interventions identified.

**Table 4:** The nature and characteristics of the interventions

Type of intervention	N	References
**Psychosocial education**		
Teaching/education	6	[27–32]
Educational workshops	3	[28, 29, 32]
**Adherence counselling**		
Group counselling/ support groups	5	[27–29, 31, 32]
Individual counselling	5	[27, 29–32]
**Family centered**		
Family based psychosocial intervention	2	[27, 32]
**Fast track/fast lane services**		
Priority clinic scheduling	3	[29, 30, 32]
**Use of reminder cards/sms**		
Reminder cards/sms	1	[32]
**Intervention agent**		
Social worker	2	[28, 30]
Adolescent care provider	3	[29, 31, 32]
Clinical psychologist/Bachelor-level counsellor	2	[27, 30]
General practitioner/Nurse	3	[27, 29, 32]
Peer counsellor	4	[28, 29, 31, 32]
Lay counsellor/CHW	2	[27, 32]
**Point of intervention delivery**		
Facility-based	6	[27–32]
Community-based	2	[28, 31]
School-based	1	[29]
**Components of intervention**		
Knowledge/education on HIV/AIDS	6	[27–32]
Adherence to treatment and retention in care	6	[27–32]
Family-focused programme	2	[27, 32]
Scheduled visits	3	[29, 30, 32]
Emotional/Affective support	4	[27, 30–32]
Structural support (youth clinic)	4	[28, 29, 31, 32]
Sexual and reproductive health	5	[27–31]
Disclosure, stigma and discrimination	5	[27–29, 31, 32]
Health promotion	1	[32]
AIDS related loss and bereavement care	1	[27]

Health and psychosocial education delivered in the form of educational activities and workshops to provide information on HIV and other relevant topics formed an integral part of all six interventions [27–32]. Health education was delivered using posters and cartoons in a structured manner that provided participants with real life situations on navigating through being orphaned by AIDS; moving in with relatives; learning about own HIV diagnosis and treatment needs, while coping with family loss, stigma, peer relationships, identity, and family functioning [27–32]. Furthermore, trained staff who were equipped with the tools to care for and skilled in treating adolescents were employed in six of the interventions e.g. adolescent care providers, youth-focused social workers and psychologist [27–32].

Six studies evaluated interventions that involved individual and peer counselling as part of the interventions [27–32]. The counselling sessions were facilitated by trained community adolescent treatment supporters (CATS), social workers, lay health workers, trained health professionals, or research teams, and aimed to increase HIV knowledge and address adherence and retention barriers [27–32]. These individual counselling methods used a client-centred approach [27, 30–32], or motivational interviewing [28] or peer counselling [29]. Group counselling or support groups were found in five articles as a means of psychosocial support [27–29, 31, 32]. Youth specific support groups addressed issues such as emotional needs; developing self-management skills; capacity building; sexual health; and the stigma related to HIV [28, 31, 32].

One study implemented a school-based programme to create a supportive environment for adherence for YPLWH [29]. The programme offered counselling at schools on sexual and reproductive health and encouraged adolescents to establish health clubs among themselves [29]. In addition, the intervention provided HIV medication on the school premises to enhance adherence and linkage to care, as well supporting participating learners in disclosure [29].

Two of the interventions had family-centred services [27, 32]; with one intervention implementing a family clinic day (FCD) [32]. FCD applied to paediatric and adolescents living with HIV and their immediate family who received priority HIV-care and counselling on a day allocated specifically to them [32]. Another component of FCD was the use of reminder cards and calendars for scheduling appointments. Health education workshops were held, which were led by peers equipped in leading discussions around HIV, sexual and reproductive health, adherence, disclosure, puberty and life skills [27–29, 32]. In addition, the Vuka family programme [27] another family-centred intervention conducted 10 health education workshop sessions that covered subject areas addressing mental and depressive disorders experienced by adolescents living with HIV. These sessions included AIDS-related loss and bereavement, HIV transmission and treatment knowledge; disclosure of HIV status to others; youth identity, acceptance, and coping with HIV; adherence to medical treatment; stigma and discrimination; caregiver child communication, particularly on sensitive topics such as puberty and HIV. The Vuka family programme also identified and developed strategies to keep children safe in high-risk situations where sexual behaviour and drug use are common [27]. Furthermore, integrated group sessions were held that were comprised of HIV-infected youth and their caregiver/s, as well as separate group activities for caregivers and preadolescents.

In the case of the Red-Carpet Intervention, adolescents were given VIP express cards- a card offering adolescents fast-track counselling and HIV treatment [29]. One of the interventions also offered adolescents the opportunity to schedule their appointments [32]. Moreover, adolescent waiting areas were implemented to create an adolescent-friendly environment aimed at improving retention to ART services at facilities [28, 32]. Although referral systems were used in two of the programmes, the programmes lacked the services needed by participants, like individual counselling [27]; and support groups for substance abusers; and housing or nutrition services.

Reminders cards and sms were used in one of the studies [32]. Participants were scheduled to attend their next appointment visit using reminder cards and reminded to take their medications by sending SMS messages at regular intervals.

### Outcomes measured

The primary outcomes of interest were adherence to ART and retention in care. The measures of psychosocial support outcomes reported were: (i) self-management (self-efficacy and self-esteem), which is associated with improved self-concept and future orientation [27, 28, 31, 32]; (ii) reduction of stigma and discrimination [27, 31]; (iii) disclosure and communication [27, 31, 32]; and (iv) perceived support in the form of social support, instrumental support, family and/or peer support and informational support [28–32]. Our findings showed that five of the studies [28–32] reported on both the primary and secondary intended outcomes [Table 5].

**Table 5:** Reporting of primary and secondary intervention outcomes

Outcomes	Studies					
	[30]	[32]	[28]	[29]	[27]	[31]
Retention in care	**✓**	✖	**✓**	✖		**✓**
Adherence to medication		**✓**	✖	✖	**✓**	**✓**
Self-management					**✓**	**✓**
Disclosure/Communication		**✓**			**✓**	**✓**
Social support					**✓**	
Instrumental support	**✓**			✖		
Family and/or peer support		**✓**		✖		**✓**
Information				✖	**✓**	**✓**
Confidence, self-esteem, self-worth						**✓**

**✓**: Reported statistical significance

✖: No statistical significance

Reference [28] only reported outcomes of retention in care and adherence to medication and no other psychosocial outcome. Nevertheless, the reference was included because its intervention included educational activities and support groups offered by social services staff trained in the use of motivational interviewing

Retention in care was investigated in five of the six studies [28–32]. Three studies found retention in care at 24 months [28], 12 months [31], and 6 months [30] to be significantly higher following exposure to the psychosocial interventions. Wohl et al. [30] found that participants’ HIV clinic visits significantly increased between baseline and at 6 months following the youth case management intervention (*p* < 0.0001). Davila et al. [28] found that the centralisation of youth services, which was composed of multifaceted psychosocial intervention components, improved the retention in care of YPLWH (*p* < 0.01) at 12 months. However, there were no significant differences observed in baseline viral load by service era (*p* = 0.91) [28].

Similarly, Ruria et al. [29] conducted a pre- and post-intervention to measure retention of YPLWH in ART care. The findings indicated that after 1 month, 90% of patients were linked to care in the pre-intervention cohort compared to 85.7% in the post-intervention cohort. The high rate of linkage to care in the pre-intervention phase was attributed to the national policy on Adolescent Reproductive Health and development [29]. However, the results show that following the implementation of the peer counselling and psychosocial support intervention, a significant increase from 66 to 90%; and 54.4 to 98.6% were observed at 3 months and 6 months respectively. While there is a high rate of YPLWH linking to care within the first month of ART initiation, these numbers dropped with time, and the intervention better success in linking YPLWH to ART over time.

Results from the Family Clinic Day (FCD) intervention showed a significant increase in patient adherence to clinic appointment schedules, that is 65% (*p* < 0.01) of adolescent participants were adherent to their appointment schedules compared to 53% participants in the control facilities). However, no effect on retention in care between the control group and the intervention group (*p* = 0.94) was observed [33].

Adherence to medication was reported as a significant outcome in three studies [27, 31, 32]. According to Bhana et al. [27], a self-reported scale on how often medication was missed over the past 6 months by participants in the VUKA intervention reported significantly greater adherence to treatment than those in the control group (*p* < 0.05) [27]. Willis et al. [31] found that the intervention group were 3.9 times more likely to adhere to treatment compared to the control group.

Four of the studies reported on secondary outcomes [27, 30–32]. The study conducted by Wohl et al. [30] showed that personalised case management interventions provided instrumental support for participants (tangible help provided by others). For example, support in the form of referrals for housing, mental health services, risk reduction education and transportation assistance within the first 6 months post the intervention [30]. Similarly, qualitative findings from the FCD intervention conducted by Graves et al., suggests that the family groups component of the intervention provided participants with increased instrumental, family, peer, and informational support [32]. The findings from the VUKA pilot programme reported significant increases in individual self-concept and future orientation, improved parent-child communication, improved social support and informational support [27]. Furthermore, caregivers reported improved family support, and a decrease in the experience of stigma [27]. One study investigated the effects of community adolescent treatment supporters on psychosocial wellbeing [31]. Willis et al. [31] found a statistically significant increase in confidence, self-esteem and self-worth (*p* < 0.001). In addition, the intervention group reported a statistically significant improvement in the quality of life, while the control group reported a significant decline in the quality of life (*p* = 0.028) [31].

## Discussion

Our review revealed that individual and peer counselling was a distinctive treatment modality when focusing on improving ART adherence, linkage to care and/or retention in care [27–32]. While in two instances, individual counselling was carried out using client-centred theory [30] and motivational interviewing [28], one study employed trained community adolescent treatment supporters (CATS) to provide peer to peer support to YPLWH [31]. These techniques have proved to improve adherence and retention in care among YPLWH [22, 31]. Motivational interviewing is confirmed to help people adopt better health behaviours such as helping young people to use condoms more often, and also to reduce viral load [34]. Individual counselling interventions have also been identified as resource-intensive approaches [16] as they are applied at an individual level. Individual-focused counselling are labour-intensive and thus challenging to implement in low and middle-income countries (LMICs). However, equipping low cadre health care workers such as peer lay counsellors or CATS with the necessary skills could prove effective in providing ART care and support tailored to adolescent’s particular needs [35].

Support groups were used in five of the interventions [27–29, 31, 32], whereby a space (physical and/or psychological) was created for participants to share knowledge, build social capital and expand their support systems. This method of delivering psychosocial support has been found to improve adherence, linkage to care and quality of life, thus constituting a viable treatment option in LMIC where healthcare staff and resources are limited [28]. Peer support has been reported as a major source of social support and information among adolescents in relation to living with HIV [29, 31]. Furthermore, centralising health services for youth have the propensity to reduce barriers to retention and adherence to ART care by providing medical and social services at one central location and reducing the need for navigating complex healthcare systems and improving coordination of services. The enhanced centralised youth service programme attempted to reduce negative health beliefs and misinformation about HIV by supporting patients’ emotional needs and providing youth friendly HIV education to address misconceptions about living with HIV [28, 30, 31]. Youth specific support groups and educational activities offer opportunities for young people to develop support systems, knowledge, and self-management skills.

Family/household-centred services were found in two articles [27, 32], which enhanced family cohesion and communication in both cases. The family/household-centred care approach argues that the family shares the responsibility of caring for the YPLWH [36]. A recent review conducted to explore the availability and effectiveness of family/household-focused interventions to improve ART adherence and retention in care found that some of the HIV-related interventions with a household focus were focused on YPLWH, and incorporated aspects of information sharing on HIV; improving communication; stimulating social support and promoting mental health [33]. Furthermore, studies have shown that integrating paediatric and adult services has positive outcomes on adherence and retention in care [37]. Additionally, the VUKA family programme addressed sensitive topics relating to HIV by using a culturally tailored cartoon [27]; such interactive modes of delivering interventions have been found to enable parent-child communication [38].

Appointment cards were used in one of the interventions where calendars and reminder cards helped schedule eligible patients to attend their next appointment on a family clinic day [32]. There is growing evidence from published literature that mHealth as a means of active client follow-up could improve the retention of patients in care through sending of SMS reminders of their appointment dates [39, 40]. The World Health Organization recommend using mobile phone reminders to improve adherence, bearing in mind that the process should be carefully monitored when aimed at adolescents for effective implementation [41]. In addition, it has been argued that adherence interventions adopting a single approach, such as phone call reminders, are less effective compared to multicomponent interventions that mobilise several support strategies and delivery modalities [42], specifically due to lower cell phone network coverage in rural and remote areas in LMICs [43].

Our scoping review identified six studies that reported on the effects of psychosocial interventions on adolescent adherence to ART and retention in care. Despite the growing recognition of the burden of HIV and psychosocial challenges faced by YPLWH, this review indicates that there is a dearth of evidence on psychosocial support interventions aimed at YPLWH. Other authors have shared the same sentiments [44, 45]. Strasser et al. [44] state that evidence-based psychosocial support services for children are currently under-developed and under-resourced, and argue that the current state of affairs need to be addressed and improved. Petersen et al. [45] also identified the need for targeted efficacy-based mental health promotion interventions for children and adolescent HIV populations in South Africa.

Five studies in this review reported increased retention and adherence to ART among adolescents and young people following the administration of an intervention with psychosocial components [27, 28, 30–32]. A study evaluating the effects of a psychosocial intervention among PLHIV attending clinical care in Estonia reported that the intervention increased the proportion of patients that were optimally adherent [46]. Similarly, a study conducted by Tominari et al. [47] reported that the implementation of mental health services demonstrated a significant increase in retention in care among PLHIV.

Evidence suggests that ART adherence interventions need to adopt long-term and flexible approaches to effectively support adherence behaviour [42]. The study conducted by Wohl et al. [30] reported that a significant dose response trend was observed between retention in care and increasing number of hours in the intervention and increasing number of intervention appointments.

Furthermore, Wohl et al. [30] found that a time-intensive intervention delivered by a non-judgemental and culturally competent peer is effective in engaging participant in consistent ART care. These findings are supported by previous studies, which suggest that intensive interventions are required to produce effective adherence outcomes, while one-time interventions without ongoing educational support may prove inefficient [48]. According to Edwards and Barker [49], developing frameworks for understanding and describing contexts, which incorporate an adaptive approach for intervention implementation and scale-up are necessary to advance HIV/AIDS implementation research and to ensure the effectiveness of an intervention.

We learnt from the scoping review that psychosocial support interventions for YPLWH are feasible and acceptable to participants and healthcare workers. However, more empirical evidence is needed to understand the mechanisms which allow these interventions to work, to improve the availability of services and care for YPLWH. Limited information exists regarding the effectiveness of adherence interventions for YPLWH in LMICs [1]. The findings from the CATS and VUKA programme indicate that psychosocial interventions may be successfully implemented to improve YPLWH adherence to ART in resource limited settings. These findings are supported by a recent study reporting on the effectiveness of teen adherence clubs in Zimbabwe and South Africa [50].

### Limitations and strengths of the review

A strength of this scoping review is our extensive and comprehensive database search that encompasses global peer reviewed papers with a narrative reporting approach. All questions related to inclusion/exclusion of a study were discussed with the investigating team. We observed significant heterogeneity in measurements and definition of optimal adherence and inclusion criteria for participants in the different studies.

The limitation of this scoping review and inference of results is limited by the quality of the individual papers underlying the process. For example, many of the papers included had small sample sizes. Further limitation to this scoping review is the exclusion of interventions that may have been evaluated using qualitative methods such as those conducted by Dorothy et al. [51], Donenberg et al. [52] and Mahvu et al. [53]. In addition, we only focused on English publications and those published after 2004 introducing the potential to have excluded studies that might have otherwise met these inclusion criteria. Our focus in the last 15 years was meant to capture the most recent evidence because so much has changed in the HIV/AIDS treatment and care protocol since its inception. Capturing the last 15 years would provide more relevant evidence regarding the most recent treatment care protocols. Furthermore, self-reported measures are fraught with bias compared to more objective measures of adherence such as viral load, antiretroviral drug levels and pill counts. Lastly, in this review, we did not differentiate the impact of behavioural patterns as a result of the intervention offered. For example, exploring the behavioural patterns between newly acquired HIV vs perinatal HIV. We also did not delineate the different age groups 10–19 years and 20–24 years as these age groups’ psychosocial needs are different.

## Conclusion

Individual and group counselling including family-centered group counselling and the use of adolescent peer support were distinctive treatment modalities when focusing on improving ART adherence, linkage to care and/or retention in care. However, this review found only six studies that evaluated psychosocial support interventions suggesting that there is dearth of evidence on psychosocial support interventions to improve adherence and retention in ART care amongst young people living with HIV. Where studies exist; methodological quality, target population, and sample size are limited. Future research and programming should seek to address psychosocial support interventions or approaches specifically designed to address the needs of YPLHIV. 

### Appraisal of the study quality

Methodological appraisal of study quality or risk of bias of the included articles, was based on the criteria provided by the NIH-NHLBI Quality Assessment of Systematic Reviews and Meta-Analyses assessment tool.

### Reporting

The scoping review was reported following the PRISMA guidelines to enhance transparency in reporting.

## Supplementary Information

**Additional file 1.**

## Data Availability

Because the study was a scoping review of published studies, the full references of these studies have been provided in the reference list.

## Abbreviations

ART: Antiretroviral therapy
HIV: Human Immunodeficiency-Virus
YPLHIV: Young people living with HIV
OLHIV: Older adults living with HIV
PICOT: Population, Intervention, Comparison, Outcome and Time
PRISMA: Preferred Reporting Items for Systematic Reviews and Meta-Analyses
