# Supplementary material for: Psychosocial support interventions for improved adherence and retention in ART care for young people living with HIV (10–24 years): a scoping review
Source: BMC Public Health. 2020 Dec 1;20:1841. doi: 10.1186/s12889-020-09717-y (PMC7708133; doi:10.1186/s12889-020-09717-y)
Supplement: Supplementary file 1 — Additional file 1. [file 12889_2020_9717_MOESM1_ESM.pdf]

Author	Time period	Aim/objectives	Country	Study Design	Intervention	Participants/population/sampling	Age group	Findings relating to Psychosocial support, improvement in retention in care an adherence to ART (Detailed description of outcome)	Measures investigated (i.e. mental disorder, self-efficacy, self-esteem etc..)	Primary Outcome	Strength of evidence
Wohla*, et al, 2011	2006-2009	Youth are known to test late for HIV, delay seeking care for an HIV infection following an HIV test, are at high risk of dropping out of HIV care and have poor adherence to antiretroviral treatment regimens. Therefoe, this paper presents the evaluation of a clinic-based, youth focused case management intervention to engage and retain Latino and African-American in HIV primary care services..	US	Intervention evaluation -Pre and post study	YCM combined psychosocial case management, treatment education/adherence support, and HIV risk reduction counseling to provide a client centered intervention. The clinic-based intervention was administered by two para-professional, Bachelor-level case managers who were trained and supervised by a licensed clinical social worker to deliver the intervention in a non judgemental and culturally appropriate manner. The participants met weekly with case managers for the first two months and monthly for the next 22 months making a total of 12 case management visits. Participants were first screened to assess their medical, physical, psychosocial, environmental, and financial needs. Then using the change model participants were categorised into the respective stages of with respect to initiation and utilisation of HIV care. Subsequently, based on particpant category, the case managers and participants develop an individualized treatment plan to address identified barriers to engagement and retention in HIV care corresponding to their stage of change. Furthermore, participants were provided $25 quarterly for their participation totalli $200 for the 24 month intervention	61 Young gay men.	18-24 years	The findings showed that at baseline, 66% of the participants had depressive symptoms, with CES-D score of 16 or more. Amongst African American, 52 % reported life time drug useand 54% reported any life time drug use amongst Latinos. 46% of overall sample reported reported life time marijuana use, 13% stimulant use, 8% inhalant use, and 23% other drugs. one participant exited the programme early and seven were lost to follow-up. At the time of enrolment, 86% of African American and 71% of Latinas had a critical need for housing, nutrition, substance abuse treatment, or mental health services. Retention in HIV care at six months was associated with increased number of intervention visits (p 0.05), more hours in the intervention (p 0.02), and prescription of HAART. These data highlight the critical needs of HIV-positive African-American and Latino YMSM and demonstrate that a clinic-based YCM can be effective in stabilizing hard-to-reach clients and retaining them in consistent HIV care. The $25 incentive was also helpful in motivating clients to the appointments.	Demographic and psychosocial characteristics, sexual risk behaviours, substance abuse, depression, and HIV testing and care history. In addition, data on prescribed ART reimens, CD4 counts, and attendance to HIV care appointments were abstracted from medical records.	proportion of YMSM retained in HIV care at six months.Linkage and retention in care	Good
Davilaa, et al., 2013	2002-2008	The paper investigated the effect of clinic-wide changes in delivery of youth services on retention in HIV care. The hypothesis stated that retention in HIV care would mprove with the centralization of clinical care and addition of youth specific supportive services.	US	Retrospective cohort study	Centralisation of youth friendly clinic within the primary health care: patients were divided into three distinct service eras: namely, decentralized era, the centralized era, and the centralized era with supportive services. During the decentralized era, no youth specific HIV care programs were available. In the centralized era, youth are seen ina multi-disciplinary youth clinic that was staffed by adolescent care providers and youth-focussed social workers and case managers. Lastly, an era were enhanced youth services were offered to centralized care. The new services included a youth specific support group and youth-focussed educational activities. Case managers and social workers were also trained to use motivational interviewing to improve self-efficacy, teach healthcare navigation skills, and encourage HIV disease management.	174 patient records	13-23 years	87% of the patient records wee between 18 and 23 years old. Overall, 54% had adequate viisit constancy, including 31% during the decentralized era, 57% in the centralized era, and 65% in th centralized with enhanced service era. logistic regresion model showed that patients in the decentralized era were less likely to have adequate visit constancy compared to patients in the centralized era, while no signifcant improvement in visit constancy was observed between patients recieving care in the centralized era compared to the centralized with enhanced service era. Similarly, overall 85% of patients had no gap in care during the first 12 months following entry into HIV care. 83% of patients who entered care during the decentralized era did not have a gap compared to 80% in the centralized era, and 96% in the centralized with enhanced services era.. Patients in the centralized with enhanced services era were 5 times more likely to not have a gap in care compared to patients in centralized era. Centralization of youth services was associated with a significant increase in retention in care through improved visit constancy. During the period in which enhanced services were added, an additional increase in retention in care occurred through a reduction in patients with a gap in care. These findings suggest that centralizing youth services coupled with enhanced services may lead to improvements in retention in care in this vulnerable population.	Centralizing youth services can reduce barriers by providing medical and social services at one central location, reducing the need for navigating complex healthcare systems and improving coordination of services. Negative health beliefs and misinformation about HIV have also been found to play a role in low retention in care. The enhanced service program attempted to reduce these issues by supporting patients emotional needs and providing youth friendly HIV education to address misconceptions about living with HIV. Youth specific support groups and educational activities offer opportunities for youth to develop support systems, knowledge, and self-management skills.	Visit constancy and gaps in HIV care were the two measures used in measuring retention in HIV care.	Good
Graves et al., 2018	October 2014 to March 2015	The primary objective of this study was to determine whether the FCD intervention was able to improve the proportion of paediatric and adolescent patients that were retained in care and adherent to their treatment schedule	Uganda	RCT	Family clinic day (FCD) was designed as a differentiated care model to ensure prioritization of HIV treatment and counselling for any paediatric and adolescent patients, including their immediate family members. Following consultation with experienced counsellors, a set of resources were developed to target the educational needs specific to adolescents and caregivers of paediatric HIV patients. In addition to the intervention package, all standards of HIV care were provided to eligible patients when attending the clinic on an FCD. The FCD intervention involved three main components as part of the differentiated care model:<br>1. Patient scheduling: Any age-eligible paediatric and adolescent patient, including their family unit, was scheduled to attend the ART clinic on a regularly occurring designated day in order to re-route all such patient appointments onto the same day. Calendars and reminder cards helped schedule eligible patients to attend their next appointment on a FCD. Promotion of the FCD intervention was strictly facility-based among intervention sites.<br><br>2. Health education: Two separate specialized health education sessions were conducted during each FCD; one session targeted adolescent patients while the other was conducted with paediatric patient caregivers. Each session was led by an expert client, a long-term adherent patient, using high quality illustrated flip charts specifically aimed at increasing knowledge of HIV basics and background, adherence, disclosure, puberty, sexual and reproductive health, and life skills among other topics. FCD clients were highly encouraged to attend HIV health education sessions during FCDs.<br><br>3. Patient flow: During a designated FCD, children, adolescents and their families were prioritized for care over other patients. Patients ineligible for FCD were purposefully scheduled on non-FCD designated clinic days. Clinicians and ART staff among intervention facilities were empowered to determine their respective FCD schedules. Trainers helped guide decision-making, which was based on calendar inputs using pre-existing ART clinic schedules; facilities implementing 2 or fewer ART clinic days per week were encouraged to schedule 1 FCD per month, while all other facilities implemented FCDs on a weekly basis. Factors such as national school holiday schedules, and other clinic-based or local community-based events helped select specific days for FCD implementation. Created in collaboration with the Ugandan MOH, a standardized FCD implementation guidebook was used as the primary training tool of a two-step cascade-training course. This training was first conducted centrally for master trainers, including senior chief medical officers and senior nursing officers who then stepped down training regionally among all participating facilities. The 2-day training covered all aspects of FCD implementation including study background, FCD introduction, intervention set-up, scheduling, counseling, and record keeping.	4,504 paediatric and adolescent patient records	19 months to 19 years	The findings of this study suggest that the provision of HIV care to paediatric and adolescent patients through an FCD can play an important role to increase adherence to clinic appointment schedule. The study found evidence that paediatric and adolescent patients in FCD facilities were more likely to be adherent to their appointment schedule compared to those in non-FCD facilities, thereby having the opportunity to be adherent to ART. Further, qualitative interviews suggested an improved quality of ART services through health education and increased psychosocial support. The study observed no significant improvement in paediatric and adolescent retention, which may be due to the very high retention outcome found among study facilities and the limited follow-up period.	Family and peer psychosocial support: Within family groups, participants discussed a number of improvements in the relationships and support networks as a result of the FCD program. First, that the FCD program had facilitated disclosure of HIV status within the family unit. Previously, caregivers were collecting drugs at the facility, but health education about HIV status disclosure to a child had never been discussed so children did not understand the purpose of medication. In some cases, adult members of the same family were collecting drugs from different facilities and had not disclosed status to each other. Thanks to improved disclosure of HIV status in the FCD intervention, family members could remind each other to take medications and adhere to appointment schedules. Also, within families the FCD program may have helped engaging certain types of family [illegible]	Included proportion of patients retained in care at 6 monthsand the proportion adherent to their appointment schedule at last study period scheduled visit. Patients were classified retained in care if they attended any ART clinic appointment at least once over the last 3 months of the study period.	Fair
Bhana, et al., 2014		A pilot RCT of VUKA was conducted to examine the (1) development, feasibility, and acceptability of VUKA for health care settings in South Africa and (2) short-term impact of the VUKA family program on a range of psychosocial variables for YLWPHIV and their caregivers.	South Africa	RCT-All participants were assessed at two time points, at baseline and approximately two weeks after the last intervention session (approximately three months after baseline); 59 families (91% of participants) completed the posttest assessment.	Family based psychosocial intervention: A new culturally tailored cartoon story-line and curriculum enabled lay counselors supervised by a psychologist to deliver the intervention in an engaging and structured way. The cartoon story-line tells the story of a 12-year-old boy, orphaned by AIDS, who moves in with relatives and learns about his own HIV diagnosis and treatment needs, while coping with family loss, stigma, peer relationships, identity, and family functioning. The curriculum provides step-by-step guidance for counselors to deliver critical information to facilitate discussions and problem-solving within and between families in multi-family groups. Session topics include: (1) AIDS-related loss and bereavement; (2) HIV transmission and treatment knowledge; (3) Disclosure of HIV status to others; (4) Youth identity, acceptance, and coping with HIV; (5) Adherence to medical treatment; (6) Stigma and discrimination; (7) Caregiver child communication, particularly on sensitive topics such as puberty and HIV; (8) Puberty; (9) Identifying and developing strategies to keep children safe in high-risk situations where sexual behavior and drug use are possible; and (10) Social support. Furthermore, HIV-infected youth and their primary caregiver come together with other affected families for sessions, which include both multiple family group activities and separate parent and child group activities. The intervention was administered in six sessions over a three-month period (two Saturdays a month) based on participant and provider feedback concerning feasibility and space. Intervention facilitators were primarily lay counselors and one masters-level psychologist, who also provided supervision after initial training by the study team	65 families, and 66 adolescents	10-13 years	Despite the small sample size, comparisons between VUKA and the comparison group arms revealed some significant differences and trends on key domains including reported ART adherence, HIV treatment knowledge, and caregiver child communication, with participants in the VUKA arm improving in all key outcomes. Youth in the VUKA condition evidenced significantly greater improvements in reported ART adherence than youth in the comparison group post-intervention (reported as change scores), Caregivers in VUKA reported significantly greater change in comfort communicating with their children about sensitive topics and a trend for experiencing less stigma than caregivers in the control group. There was also a trend for youth in VUKA to show more improvements in treatment knowledge , and caregiver child communication than nonintervention group youth. Further, Improved self-concept and future orientation-Caregivers reported that VUKA helped promote healthy HIV-positive self-identities and future orientation, particularly through the Themba character in the storybook, and Improved social support- Caregivers reported strengthened social support for both themselves and their children through new friendships with other HIV children and their caregivers:	The measures used assessed youth adherence to ART, youth mental health; child depression; self-concept;youth treatment knowledge; HIV/AIDS illness stigma; Youth care giver communication and comfort; andHIV/AIDS illness stigma	Adherence to ART; youth adherence to ART, youth mental health; child depression; self-concept;youth treatment knowledge; HIV/AIDS illness stigma; Youth care giver communication and comfort; andHIV/AIDS illness stigma	Good

Ruriaa, et al., 2017	August 2016 to October 2016	The aim Kenya red carpet program (RCP) was to develop, implement, and evaluate a comprehensive, fast-track, peer-designed linkage-to-care and early retention program with interlinked facility and community- level components. The study evaluated the effect of the pilot RCP on the timing and outcomes of linkage to care and treatment, as well as early retention on ART, among adolescents and youth newly diagnosed with HIV in Homa Bay County.	Kenya	A pre and post implementation evaluation	RCP provided fast-track peer-navigated services, peer counseling, and psychosocial support at HCFs and schools in six Homa Bay subcounties in 2016. RCP training and sensitization was implemented in 50 HCFs and 25 boarding schools. schools agreed to implement treatment and adherence support services for adolescents and youth living with HIV in secondary boarding schools and improve linkage to HCFs and adolescent health services in Homa Bay. In total, 25 highvolume boarding secondary schools piloted the schoolbased support programs for adolescents and youth living with HIV, which included: counseling on HIVand sexual and reproductive health (SRH); creating a supportive environment to ensure ART adherence; creating health clubs and health education to address anti-HIV stigma; facilitating storage of HIV medications at school when necessary; supporting and providing counselling on disclosure of HIV status; and supporting linkage to HCFs. The RCP conducted a 2-day capacitybuilding workshop for 50 adolescent and youth advocates (including, but not limited to, adolescents and youth living with HIV) and guidance and counselling teachers, matrons, and nurses in 25 boarding schools. The training addressed key issues in adolescent and youth HIV support, including the Kenya Education Sector Policy on HIV/ AIDS. Peer involvement included identifying adolescents and youth living with HIV as participants for the Adolescent and Youth Peer Advisory Group (AYPAG) to inform the development and implementation of RCP services	952	15-19 and 2	The proportion of adolescent and youth clients who were linked to care increased from 56.5 to 97.3%<br>(P<0.001) (Table 1). All adolescents and youth linked to care through RCP received peer counseling and PSS (no data available for the preimplementation cohort). For the timing of linkage to care and treatment, there was no difference in the timing of linkage to care and treatment, as most adolescents and youth who were linked to care were linked within 1 month after HIV diagnosis and were initiated on treatment in both pre and postimplementation cohorts Improving linkage to care anr initiation and retention on treatment by implementing RCP. Implementation of the RCP was associated with improved linkage to care and better early retention on ART among newly identified adolescents and youth living with HIV. The timing of the linkage to carewas not significantly affected by RCP implementation	There was no clear assessment for psych	New adolescent and youth HIV diagnosis, linkage to and retention in care and treatment.	Good
Nicola willis et al	December 2014- November 2015	The study was set out to determine the effectiveness of CATS services on improving linkage to services and retention in care, adherence and psychosocial well-being among 100 ALHIV in a rural district of Zimbabwe	Zimbabwe	Randomised controlled trial	The control arm received standard of care offered by MoHCC, including monthly clinic reviews, ART, adherence counseling, CD4 monitoring and management of Ois. Tx and Care was provided by a nurse or primary counsellor.<br><br>Intervention arm provided SoC, but were also allocated trained CATS for additonal support. This included a weekly home visit during which the allocated CATS provided HIV and ART information and counselling as well as monitored the participants' adherence and general well-being.	94 ALHIV	10-15 years	The intervention group were 3.9 times more likely to adhere to treatment compared to the control group. Linkage to services and retention in care within the intervention group increased compared with a decrease in the control arm. The intervention group reported a statistically significant increase in confidence, self-esteem, self-worth ($p<0.001$) and quality of life compared ($p = 0.028$) with a decrease in the control arm.	comprised of five components, namely: demographic information; adherence; psychological well-being, and linkages and retention in care	Adherence; psychological well-being, and linkages and retention in care	Good
